# Visualizing Typical Features of Breast Fibroadenomas Using Phase-Contrast CT: An *Ex-Vivo* Study

**DOI:** 10.1371/journal.pone.0097101

**Published:** 2014-05-13

**Authors:** Susanne Grandl, Marian Willner, Julia Herzen, Anikó Sztrókay-Gaul, Doris Mayr, Sigrid D. Auweter, Alexander Hipp, Lorenz Birnbacher, Mathias Marschner, Michael Chabior, Maximilian Reiser, Franz Pfeiffer, Fabian Bamberg, Karin Hellerhoff

**Affiliations:** 1 Institute for Clinical Radiology, Ludwig-Maximilians-University Hospital, Munich, Germany; 2 Department of Physics and Institute of Medical Engineering, Technical University of Munich, Munich, Germany; 3 Institute for Materials Research, Helmholtz-Zentrum Geesthacht, Geesthacht, Germany; 4 Institute of Pathology, Ludwig-Maximilians-University Hospital Munich, Munich, Germany; AMS Biotechnology, United Kingdom

## Abstract

**Background:**

Fibroadenoma is the most common benign solid breast lesion type and a very common cause for histologic assessment. To justify a conservative therapy, a highly specific discrimination between fibroadenomas and other breast lesions is crucial. Phase-contrast imaging offers improved soft-tissue contrast and differentiability of fine structures combined with the potential of 3-dimensional imaging. In this study we assessed the potential of grating-based phase-contrast CT imaging for visualizing diagnostically relevant features of fibroadenomas.

**Materials and Methods:**

Grating-based phase-contrast CT was performed on six ex-vivo formalin-fixed breast specimens containing a fibroadenoma and three samples containing benign changes that resemble fibroadenomas using Talbot Lau interferometry and a polychromatic X-ray source. Phase-contrast and simultaneously acquired absorption-based 3D-datasets were manually matched with corresponding histological slices. The visibility of diagnostically valuable features was assessed in comparison with histology as the gold-standard.

**Results:**

In all cases, matching of grating-based phase-contrast CT images and histology was successfully completed. Grating-based phase-contrast CT showed greatly improved differentiation of fine structures and provided accurate depiction of strands of fibrous tissue within the fibroadenomas as well as of the diagnostically valuable dilated, branched ductuli of the fibroadenomas. A clear demarcation of tumor boundaries in all cases was provided by phase- but not absorption-contrast CT.

**Conclusions:**

Pending successful translation of the technology to a clinical setting and considerable reduction of the required dose, the data presented here suggest that grating-based phase-contrast CT may be used as a supplementary non-invasive diagnostic tool in breast diagnostics. Phase-contrast CT may thus contribute to the reduction of false positive findings and reduce the recall and core biopsy rate in population-based screening. Phase-contrast CT may further be used to assist during histopathological workup, offering a 3D view of the tumor and helping to identify diagnostically valuable tissue sections within large tumors.

## Introduction

Fibroadenoma is the most common breast tumor type in young women with a median age of 25 years at the time of diagnosis [1] and an incidence of 18% [2]. Together with less common benign lesions, fibroadenomas are classified as proliferative disorders without atypia, characterized by a mildly elevated risk (RR 1.3–1.9) of future malignant breast tumors [1]. In younger women, fibroadenomas usually present as palpable masses and are typically further evaluated by ultrasound [3].

Despite recent improvements in clinical breast imaging, an accurate diagnosis of fibroadenoma based on imaging alone remains challenging. In particular, population-based breast cancer screening has led to mammographic detection of an increased percentage of nonpalpable fibroadenomas, raising the need for augmented histologic assessment [3]. Furthermore, malignant breast tumors in women at high familial risk tend to express morphologically benign characteristics in conventional breast imaging such as rounded shape or sharp margins [4]. In those women, mammographic specificity is limited and up to 23% of invasive cancers appear as fibroadenoma-like masses without calcifications [5], [6]. Finally, there is a substantial overlap in clinical and imaging findings of fibroadenomas and the rare (0.3–1.0% of breast tumors) but potentially malignant cystosarcoma phyllodes [7], [8]. Phyllodes tumors may histologically prove as benign, borderline or malignant lesions with the potential of recurrence or metastasis and therefore require a different therapeutic approach including surgical wide excision, to prevent local recurrence [9], [10]. Additional specific imaging criteria for the diagnosis of fibroadenoma might therefore have the potential to spare women invasive procedures.

X-ray phase-contrast imaging (PCI) has recently been shown to increase soft tissue contrast compared to conventional absorption-based X-ray contrast, and may therefore prove particularly suited for overcoming some of the limitations in current breast imaging. While conventional X-ray techniques rely on the absorption of radiation due to differing tissue-specific attenuation coefficients, PCI is based on the phase shift, influenced by the refractive index decrement [11]. In the energy range of diagnostic imaging, the soft-tissue contrast obtained by phase-contrast techniques has been shown to be capable of outperforming the contrast achieved by absorption-based imaging [11]. A detailed review that summarizes recent research efforts in the area of PCI of the breast can be found here: [12].

Different methods have been established for PCI of the breast [11]: among those, grating-based PCI is one of the techniques applicable to conventional X-ray sources, which makes it a particularly promising candidate for future clinical implementation. Grating-interferometry provides three images simultaneously: The conventional absorption-contrast image analyzes the attenuation of X-rays within a certain tissue and depends on the tissue-specific attenuation coefficient. The differential phase-contrast image gives information about the phase shift and depends on the refractive index decrement. The dark-field signal is sensitive to small-angle scattering and thus can reveal microstructures smaller than the pixel pitch of the detector [13], [14]. Whereas the differential phase-contrast images are particularly suited to reveal soft tissue strands, the dark-field signal is able to detect microcalcifications that are too small to be resolved in conventional mammography [13], [14]. However, due to its complexity, 3-dimensional reconstruction of the dark-field signal is still under development; thus, this study concentrates on the specific value of the differential phase signal. First trials on ex vivo phase-contrast CT (PC-CT) imaging of breast samples showed greatly improved soft-tissue contrast and differentiability of fine structures compared to absorption-based imaging [15]–[17]. Microscopic structures like the walls of dilated ducts containing ductal carcinoma in situ can be visualized in PC-CT but not in absorption CT, an information that might be used for future differentiation between invasive and noninvasive carcinoma [18].

As a first study, we assessed imaging features of ex-vivo grating-based PC-CT for accurate characterization of fibroadenomas using a conventional X-ray source. In correlation with histopathology, characteristic features of fibroadenomas visible in PC-CT are described and the incremental value of grating-based PC-CT compared to absorption-based CT is outlined.

## Materials and Methods

### Study design

This prospective ex-vivo study was conducted in accordance with the Declaration of Helsinki and was approved by the institutional review board (Ethikkommission of the Ludwig-Maximilian-University, Munich, project number 240–10). All participants gave written informed consent before inclusion after adequate explanation of the study protocol. Indication to breast surgery followed recommendation of the interdisciplinary tumor conference by the University of Munich Breast Center. Inclusion criteria were a histologically proven benign breast lesion in preoperative core biopsy or a breast lesion with benign imaging features and completed preoperative conventional breast diagnostics (ultrasound, mammography).

### Preoperative diagnostics

Preoperative diagnostics included clinical breast examination, ultrasonography (ACUSON Antares Ultrasound System, Siemens, Germany), mammography (Selenia Dimensions, Hologic, USA) and sonographically-guided core needle biopsy.

Demographic parameters of all patients are listed in table 1.

**Table 1:** Patient characteristics.

Patient Nr	Age (years)	Familiar history of BC	ACR	Bi-RADS	Maximum diameter (cm)	Classification
**1**	33	Yes	IV	IV b	2.2	Fibroadenoma
**2**	30	No	II	IV	7.2	Fibroadenoma
**3**	34	No	II	IV	1.7	Cystosarcoma phyllodes
**4**	28	No	-	IV	1.6	Fibroadenoma
**5**	48	Yes	III	IV	3.0	Fibroadenoma
**6**	20	No	-	IV	5.0	Fibroadenoma
**7**	42	No	II	II	4.2	Fibroadenoma
**8**			III	II	3.0	Pseudoangiomatous stromal hyperplasia
**9**					2.0	Fibrous mastopathy

Demographic parameters of all patients (age, familiar history of breast cancer (BC)), classification of mammographic breast density according to the American College of Radiology (ACR) and preoperative imaging according to the breast imaging reporting and data system (BIRADS) as well as maximum diameter (histopathologically proven) and histological classification of the breast lesion.

**Figure 1:**
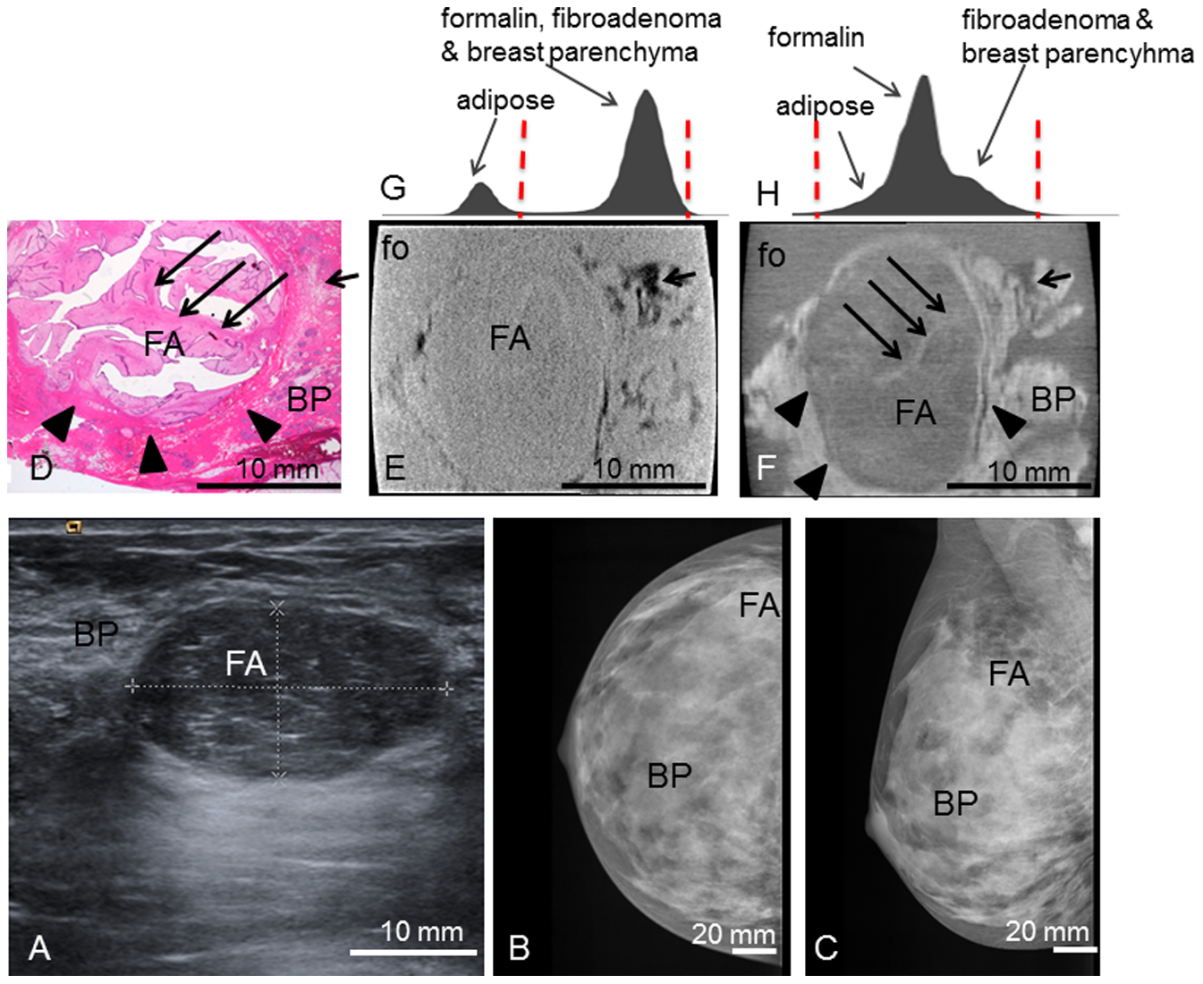
Preoperative imaging, histology, absoption- and phase-contrast CT of case 1. **Preoperative ultrasonography** (**A**) with typical benign imaging characteristics of the fibroadenoma (FA) (oval form, smooth margins, posterior enhancement); **preoperative craniocaudal** (**B**) **and mediolateral oblique** (**C**) **mammography projections** show the fibroadenoma (FA) partially hidden within the dense breast parenchyma (BP) (ACR IV). Representative **histological slice** (**D**) and corresponding **absorption-** (**E**) and **phase-contrast CT** (**F**) image. (**G**) and (**H**) show the histograms of the whole 3D volume dataset of the absorption- and phase-contrast CT, respectively: The peaks in (**H**) correspond to the different grey levels of formalin (fo), FA and surrounding breast parenchyma (BP). The “shoulder” of the histogram in (**H**) corresponds to adipose tissue. In (**G**), only two distinct peaks for adipose tissue and formalin, FA and breast parenchyma are seen. Window levels are marked with dashed red lines. The tumor boundaries are indicated by arrowheads; one sclerotic strand is indicated by long arrows. Adipose tissue is indicated by short arrows. The black sharp lines in the corners of (**E**) and (**F**) correspond to the walls of the plastic container. The ducts are artificially torn open in (**D**) due to cutting and staining procedures.

**Patient 1** presented with a palpable mass in the right breast and positive familial history of breast cancer. Clinical examination revealed an oval, mobile mass of 2.5 cm diameter in the upper right lateral quadrant. Ultrasonography (Figure 1.A) showed a smooth, oval, 1.3×2.4×2.6 cm^3^ tumor with fine internal echos and pushing margins. Mammography (Figure 1.B and C) showed a corresponding oval mass not well distinguishable within the dense breast parenchyma.

**Figure 2:**
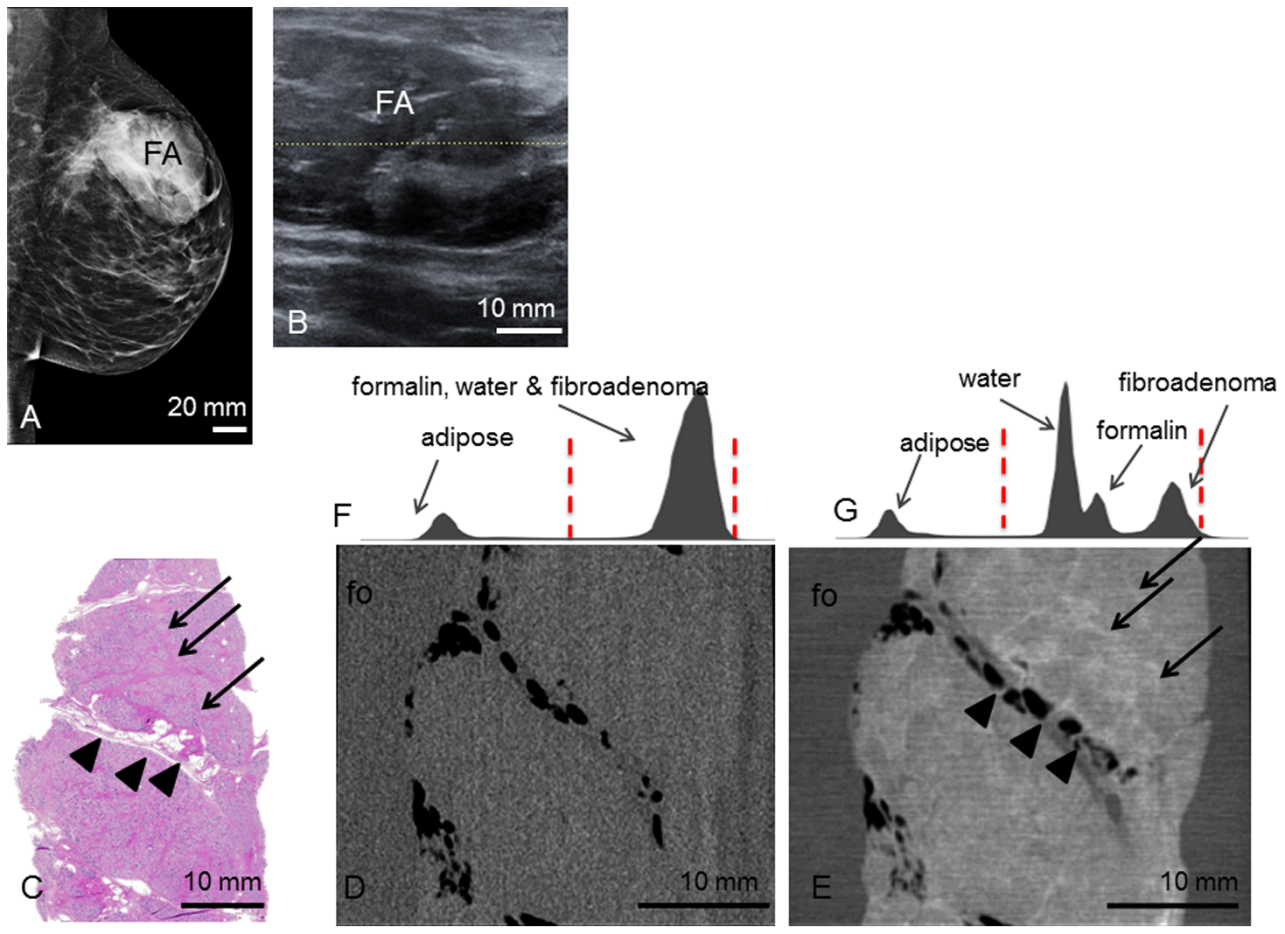
Preoperative imaging, histology, absoption- and phase-contrast CT of case 2. **Preoperative mediolateral oblique mammogram** (**A**) shows the fibroadenoma (FA) within a transparent breast (ACR II). **Ultrasonography** (**B**) of the FA. **Representative histological slice** (**C**) of the FA. Corresponding **absorption-** (**D**) and **phase-contrast CT** (**E**) slice. Arrowheads indicate septum with interspersed adipose tissue. Arrows indicate sclerotic strands. (**F**) and (**G**) show the histograms of the whole 3D volume dataset of the absorption- and phase-contrast CT, respectively. In (**F**), only two distinct peaks for adipose tissue and formalin (fo), water and FA are seen. In (**G**), there are four distinct peaks for adipose tissue, water, formalin and FA. Window levels are marked with dashed red lines.

**Patient 2** presented with a progredient swelling in the left breast. Clinical examination revealed a mobile mass of 8 cm diameter in the left upper lateral quadrant. Mammography (Figure 2.A) and ultrasonography (Figure 2.B) revealed a smooth, oval mass in the upper left lateral quadrant. The tumor measured 7.2×4.9×3.5 cm^3^ sonographically.

**Figure 3:**
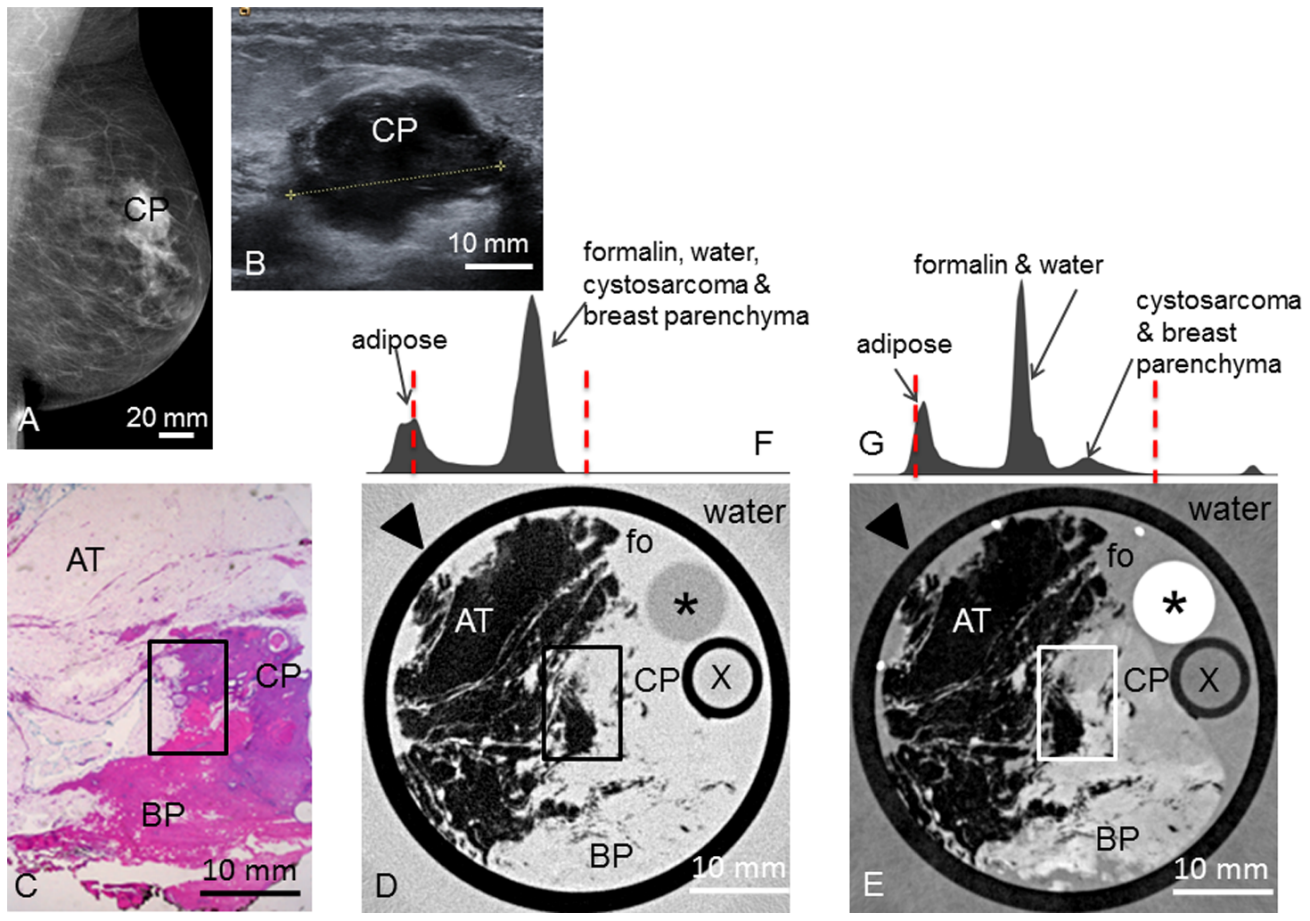
Preoperative imaging, histology, absoption- and phase-contrast CT of case 3. **Preoperative mediolateral oblique mammogram** (**A**) shows the cystosarcoma phyllodes (CP) within a transparent breast (ACR II). **Ultrasonography** (**B**) of the CP. **Representative histological slice** (**C**) of the CP. Corresponding **absorption-** (**D**) and **phase-contrast CT** (**E**) slice. Rectangle in (**C**), (**D**) and (**E**) indicates the borders between adipose tissue (AT), CP and breast parenchyma (BP). (**F**) and (**G**) show the histograms of the whole 3D volume dataset of the absorption- and phase-contrast CT, respectively, with only two distinct peaks for adipose tissue and formalin (fo), water, CP and BP in (**F**) and different grey levels for adipose tissue, formalin & water, CP & BP in (**G**). Window levels are marked with dashed red lines. Arrowheads in (**D**) and (**E**) indicate plastic container surrounding the sample. Asterisk (*) indicates polymethylmethacrylate (PMMA) stick, X indicates plastic tube filled with water; both materials can be used for calibration in quantitative studies (not used here).

**Patient 3** presented with a palpable nodule in the left breast. Palpation revealed a mobile mass of 2.0 cm between the upper left quadrants. Mammography (Figure 3.A) revealed an irregular mass between the upper left quadrants of 2.8×2.0×1.8 cm^3^. Ultrasonography (Figure 3.B) showed a corresponding irregular, hypoechogenic mass of 2.2×2.0×1.5 cm^3^ with peripheral attenuation phenomenon.

**Figure 4:**
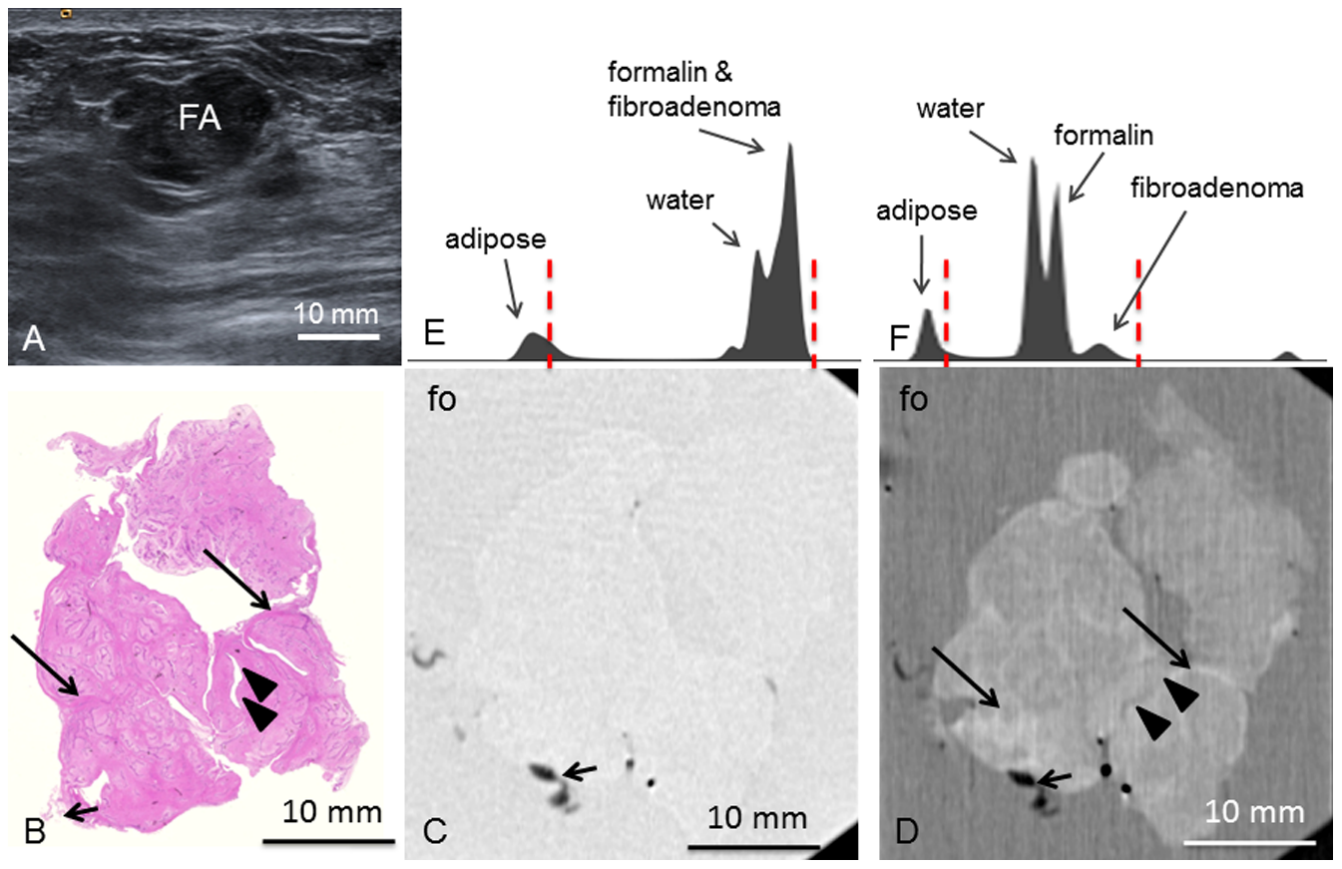
Preoperative imaging, histology, absorption- and phase-contrast CT of case 4. **Preoperative ultrasonography** (**A**) shows the fibroadenoma (FA) as lobulated mass with smooth margins. **Representative histological slice** (**B**) and corresponding **absorption-** (**C**) and **phase-contrast CT** slice (**D**). (**D**) show strands of hypocellular sclerosis (long arrows) as well as torn-open ducts (arrowheads) filled with formalin. Adhering adipose tissue indicated by short arrow. **Histograms** of the whole 3D volume dataset of the absorption- (**E**) and phase-contrast (**F**) CT, respectively, show distinct peaks for adipose tissue, water, formalin (fo) and FA in (**F**) whereas in (**E**), the peaks for water, formalin and FA overlap. Window levels are marked with dashed red lines.

**Patient 4** presented with palpable nodules in both mammae. In the right breast, a mobile nodule of 1 cm diameter between the lateral quadrants was found. Ultrasound (Figure 4.A) revealed a polylobulated, hypoechogenic mass of 1.6 cm maximum diameter with pushing margins.

**Figure 5:**
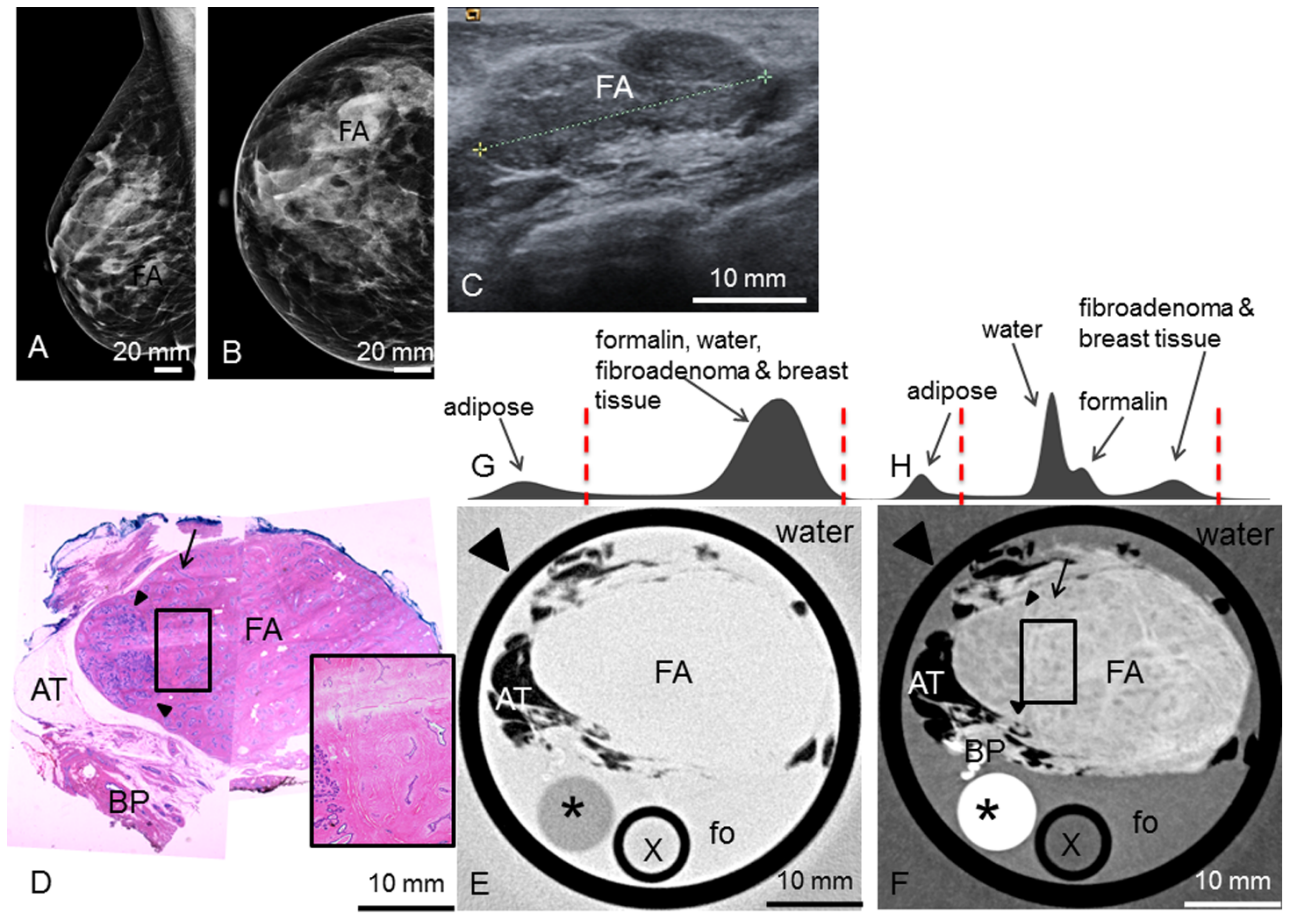
Preoperative imaging, histology, absorption- and phase-contrast CT of case 5. **Preoperative mediolateral-oblique** (**A**) **and craniocaudal** (**B**) **mammogram and ultrasonography** (**C**) showing the fibroadenoma (FA). **Representative histological slice** (**D**) showing the FA, surrounded by adipose tissue (AT) and breast parenchyma (BP). **Absorption-** (**E**) and **phase-contrast CT** (**F**) slice; black rectangle showing zoomed view of a polygonal sclerotic frame (pink in (**D**), bright in (**F**) filled with basophilic branched ducts; one linear duct indicated by arrow in (**D**) and (**F**). (**G**) and (**H**) show the histograms of the whole 3D volume dataset of the absorption- and phase-contrast CT, respectively, with only two distinct peaks for adipose tissue and formalin (fo), water, fibroadenoma and breast tissue in (**G**) and different grey levels for adipose tissue, water, formalin and fibroadenoma & breast tissue in (**H**). Window levels are marked with dashed red lines. Arrowheads in (**E**) and (**F**) indicate plastic container surrounding the sample. Asterisk (*) indicates polymethylmethacrylate (PMMA) stick, X indicates plastic tube filled with water; both materials can be used for calibration in quantitative studies (not used here).

**Patient 5** presented with a histologically proven fibroadenoma of the right breast and positive familial history of breast cancer. The mass in the lower right lateral quadrant was mobile and smooth. Mammography (Figure 5.A and B) revealed an ill-defined mass within the relatively dense breast parenchyma in the right lower lateral quadrant. Sonographically (Figure 5.C), the fibroadenoma appeared as a polylobulated hypoechogenic, smooth mass with a maximum diameter of 2.5 cm.

**Figure 6:**
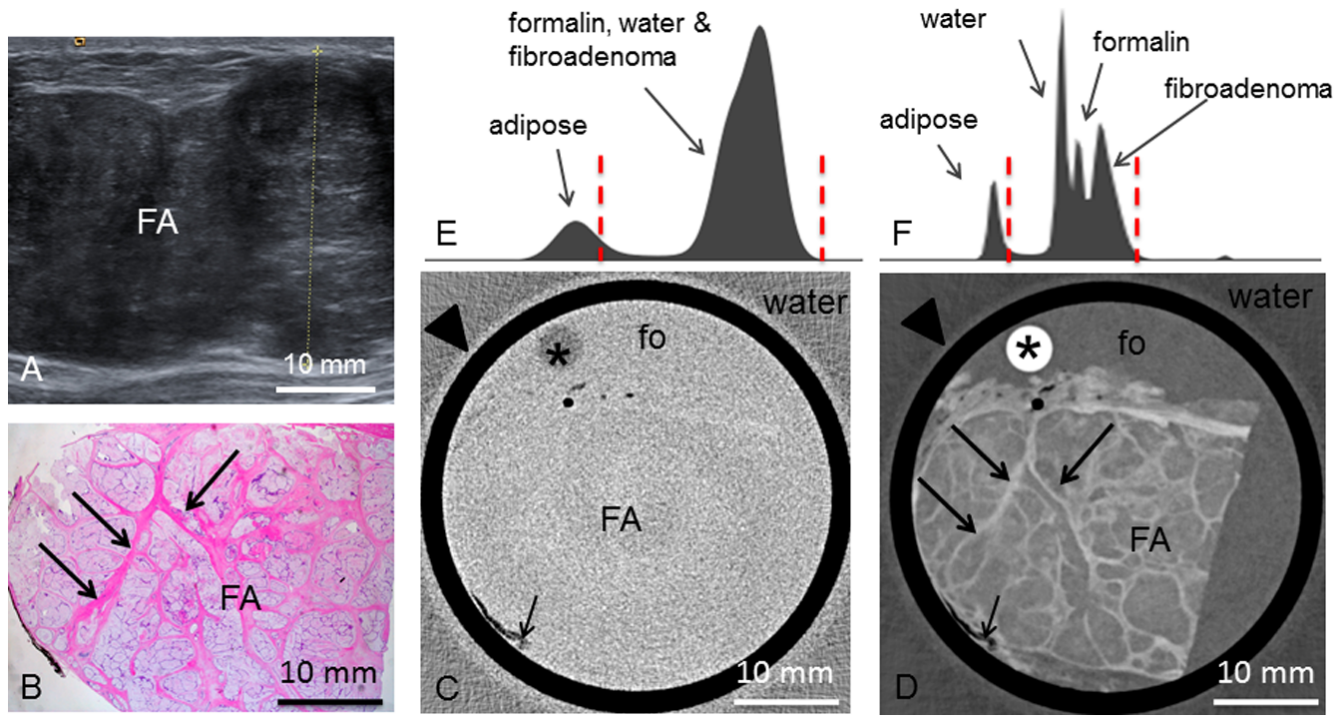
Preoperative imaging, histology, absorption- and phase-contrast CT of case 6. **Preoperative ultrasonography** (**A**) of the fibroadenoma (FA). **Representative histological slice** (**B**) of the FA. Corresponding **absorption-** (**C**) and **phase-contrast CT** (**D**) slice. Long arrows indicate strands of fibrous tissue. Short arrows indicate adhering adipose tissue. (**E**) and (**F**) show the histograms of the whole 3D volume dataset of the absorption- and phase-contrast CT, respectively. In (**E**), only two distinct peaks for adipose tissue and formalin (fo), water and FA are seen. whereas in (**F**), the histogram reveals four distinct peaks for adipose tissue, water, formalin and fibroadenoma. Window levels are marked with dashed red lines. Arrowheads in (**C**) and (**D**) indicate plastic container surrounding the sample.

**Patient 6** presented with a painful nodule in the left upper lateral quadrant that was confirmed in the clinical examination. Sonographically (Figure 6.A) a lobulated poor echogenic nodule of 5×3.7 cm with pushing margins and without posterior enhancement or attenuation was seen.

**Figure 7:**
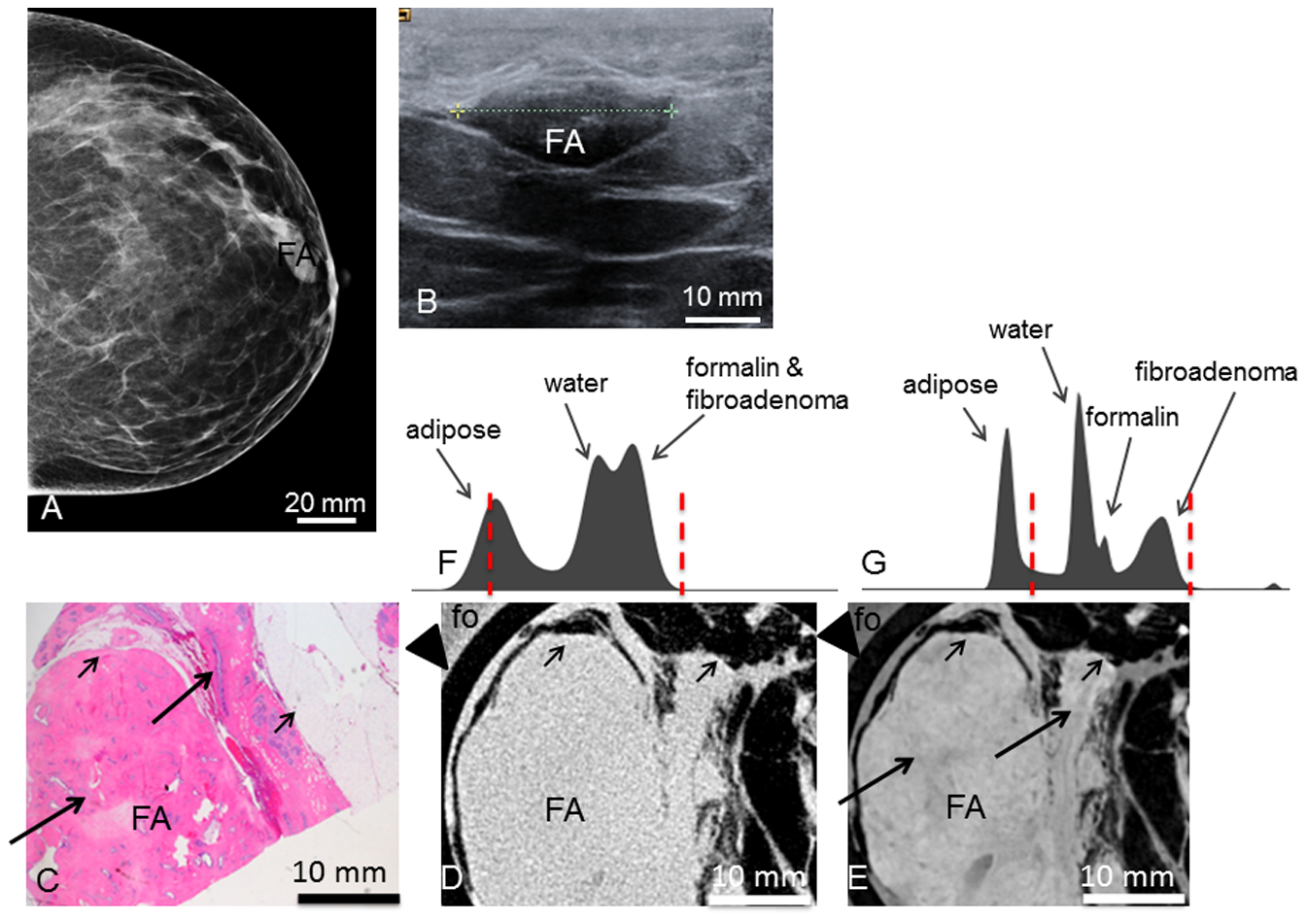
Preoperative imaging, histology, absorption- and phase-contrast CT of case 7.I. **Preoperative craniocaudal mammography** (**A**) and **ultrasonography** (**B**) of the fibroadenoma (FA). **Representative histological slice** (**C**) of the FA. Corresponding **absorption-** (**D**) and **phase-contrast CT** (**E**) slice. Long arrows indicating ducts. Short arrows indicate adhering adipose tissue. (**F**) and (**G**) show the histograms of the whole 3D volume dataset of the absorption- and phase-contrast CT, respectively. In (**F**), only two distinct peaks for adipose tissue and water, formalin (fo) and FA are seen. In (**G**), there are four distinct peaks for adipose tissue, water, formalin and FA. Window levels are marked with dashed red lines. Arrowheads in (**D**) and (**E**) indicate plastic container surrounding the sample.

**Figure 8:**
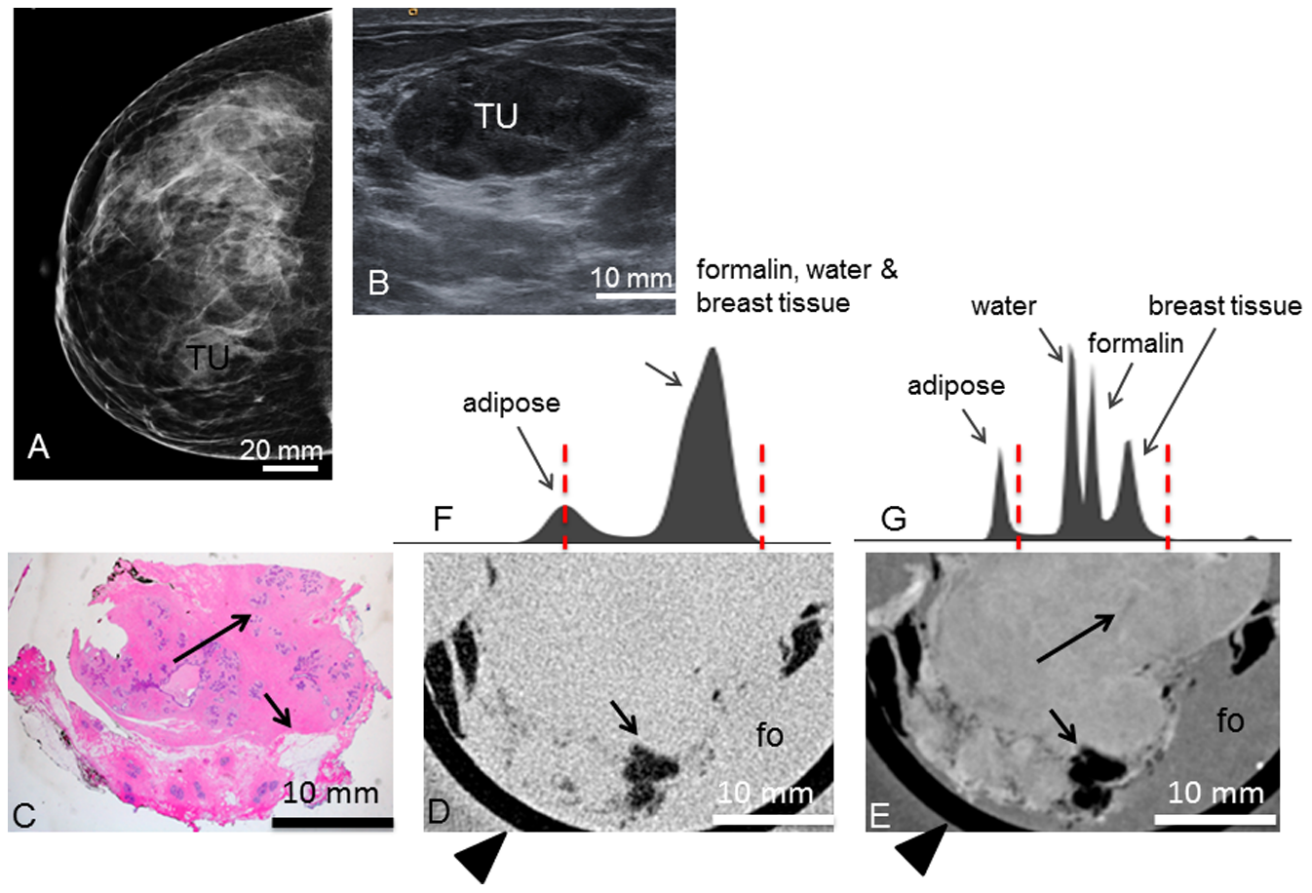
Preoperative imaging, histology, absorption- and phase-contrast CT of case 8. **Preoperative craniocaudal mammography** (**A**) and **ultrasonography** (**B**) of the tumor (TU) containing a pseudoangiomatous stromal hyperplasia. **Representative histological slice** (**C**) of the tumor. Corresponding **absorption-** (**D**) and **phase-contrast CT** (**E**) slice. Long arrows indicating ducts. Short arrows indicate adhering adipose tissue. (**F**) and (**G**) show the histograms of the whole 3D volume dataset of the absorption- and phase-contrast CT, respectively. In (**F**), only two distinct peaks for adipose tissue and formalin, water and breast tissue are seen. In (**G**), there are four distinct peaks for adipose tissue, water, formalin and breast tissue. Window levels are marked with dashed red lines. Arrowheads in (**D**) and (**E**) indicate plastic container surrounding the sample.

**Figure 9:**
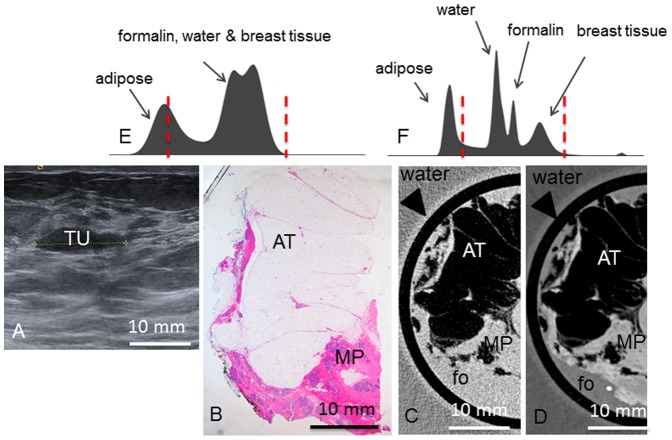
Preoperative imaging, histology, absorption- and phase-contrast CT of case 9. **Preoperative ultrasonography** (**A**) of the tumor (TU). **Representative histological slice** (**B**) of the mastopathic area (MP with adhering adipose tissue (AT). Corresponding **absorption-** (**C**) and **phase-contrast CT** (**D**) slice. (**E**) and (**F**) show the histograms of the whole 3D volume dataset of the absorption- and phase-contrast CT, respectively. In (**E**), only two distinct peaks for adipose tissue and formalin (fo), water and breast tissue are seen. In (**F**), there are four distinct peaks for adipose tissue, water, formalin and breast tissue. Window levels are marked with dashed red lines. Arrowheads in (**C**) and (**D**) indicate plastic container surrounding the sample.

**Patient 7** had undergone surgery of two fibroadenomas of the right breast one year ago and presented with a new palpable nodule of the right breast. Clinical examination revealed a mass of 2 cm diameter in the right upper medial quadrant. Mammography revealed a smooth nodule of 2.7 cm in the upper left lateral quadrant [**case 7** (Figure 7.A)] as well as an oval mass in the upper right medial quadrant corresponding to the palpable mass [**case 8** (Figure 8.A)]. Sonographically, the **tumor 7** was oval with a maximum diameter of 3 cm (Figure 7.B). The **tumor 8** was hypoechogenic with 3.0×1.4 cm diameter (Figure 8.B). Another hypoechogenic tumor [**case 9**] with maximum diameter of 1.7 cm was found in the right lateral inferior quadrant (Figure 9.A).

### Sample acquisition and preparation

All breast samples were acquired by the Institute of Gynecology, Ludwig-Maximilian-University Munich. The freshly resected breast samples were macroscopically examined by an experienced pathologist. The whole sample was put into a 50 ml plastic container (diameter 3 cm) in a 4% neutral-buffered formaldehyde solution. In cases where the sample size exceeded the container diameter (sample 2, 3 and 6), a selected representative and orientable tissue section of 3 cm maximum diameter was excised for the analysis.

### Grating-based PC-CT

The underlying principles of grating-based phase-contrast imaging in general and the adaption of the method to conventional X-ray sources can be found in Weitkamp et al. [19] and Pfeiffer et al. [20].

The experimental imaging setup used for the measurements is located at the Department of Physics and Institute of Medical Engineering of the Technische Universität München. It combines a rotating Molybdenum anode X-ray tube (ENRAF Nonius), a photon-counting pixilated imaging detector (Pilatus II from Dectris, Switzerland, 487×195 pixels, 172×172 µm^2^ pixel size) and a Talbot-Lau interferometer. The interferometer consists of three gratings with periods of 5.4 µm that are installed in equal distances from each other. The first one (G0) is made of attenuating gold structures and is placed behind the source to create an array of individually coherent but mutually incoherent sources. This allows for interference effects being induced by the second grating (G1) called phase grating. The resulting pattern can then be analyzed by slightly moving the third grating (G2) perpendicular to the beam to draw conclusions on beam attenuation and refraction caused by the examined samples. The images that are acquired in this process for each radiographic projection are usually denoted as phase steps. In analogy to conventional CT, 3-dimensional datasets can finally be reconstructed by recording many projections from different angular positions over 180° or 360° and applying a filtered backprojection algorithm. In case of phase-contrast, the filter kernel has to be replaced by the imaginary Hilbert filter [21].

Due to the scarcity of suitable specimens, the setup underwent several modifications within the time period in which the present study was executed. This involved the employment of different gratings that were all fabricated and constantly advanced in quality by the Karlsruhe Institute of Technology and Microworks GmbH (Karlsruhe, Germany). The distances between the gratings were as well adjusted to further increase the visibility of the interferometer – an important performance parameter of a grating-based PC-CT system and directly associated to the achievable contrast-to-noise ratios. In addition, the quantum efficiency of the detector was improved by changing the thickness of the incorporated silicon sensor from 450 µm to 1 mm to cut down on unnecessary dose exposure of the samples and increase photon statistics.

The setup changes allowed to decrease the number of projections per tomography and the exposure time per phase step from 1200 projections and 5 s (case 2 and 3) down to 800 projections and 3 s (case 6–9) while at the same time reducing image noise by a factor of 2.

The tube current was set to 70 mA and a tube voltage of either 35 kVp or 40 kVp was applied during data acquisition. The choice for each setup configuration was always a trade-off between higher visibility (35 kVp) and more flux (40 kVp). The setup specifications and scan parameters of all measurements are listed in table 2.

**Table 2:** Setup specifications and scan parameters.

Case number	1	2	3	4	5	6	7	8	9
Tube voltage	35 kV	40 kV	40 kV	40 kV	35 kV	40 kV	40 kV	40 kV	40 kV
Projections	1200	1200	1200	600	1200	800	800	800	800
Angular range	360°	360°	360°	180°	360°	360°	360°	360°	360°
Phase Steps	11	11	11	11	11	11	11	11	11
Exposure time/step	5 s	5 s	5 s	5 s	2 s	3 s	3 s	3 s	3 s
Photon counts/step	∼3140	∼5500	∼4200	∼4750	∼990	∼1300	∼1300	∼1300	∼1300
Visibility	∼13,5%	∼10,8%	∼11,6%	∼11,2%	∼21,2%	∼27,8%	∼28%	∼28%	∼28%
Water bath	no	yes	yes	yes	yes	yes	yes	yes	yes
Effective pixel size	100 µm	100 µm	100 µm	100 µm	100 µm	100 µm	100 µm	100 µm	100 µm
Si-sensor thickness	450 µm	1 mm	1 mm	1 mm	1 mm	1 mm	1 mm	1 mm	1 mm
Scan time	∼32 h	∼32 h	∼32 h	∼16 h	∼17 h	∼16 h	∼16 h	∼16 h	∼16 h
G0 height (material)	55 µm (gold)	55 µm (gold)	55 µm (gold)	55 µm (gold)	55 µm (gold)	70 µm (gold)	70 µm (gold)	70 µm (gold)	70 µm (gold)
G1 height (material)	8,5 µm (nickel)	8,5 µm (nickel)	8,5 µm (nickel)	8,5 µm (nickel)	9,5 µm (nickel)	5,2 µm (gold)	5,2 µm (gold)	5,2 µm (gold)	5,2 µm (gold)
G2 height (material)	55 µm (gold)	55 µm (gold)	65 µm (gold)	65 µm (gold)	65 µm (gold)	65 µm (gold)	65 µm (gold)	65 µm (gold)	65 µm (gold)
Distance G0 to G1	87,5 cm	80 cm	80 cm	80 cm	80 cm	85,7 cm	85,7 cm	85,7 cm	85,7 cm
Distance G1 to G2	87,5 cm	80 cm	80 cm	80 cm	80 cm	85,7 cm	85,7 cm	85,7 cm	85,7 cm

Setup parameters for image acquisition of each sample.

The sample containers were mounted directly in front of the phase grating and all but the first one were surrounded by water during the CT scan. The water bath was introduced to avoid the occurrence of too strong signals at the edges of the plastic cylinders that can degrade image quality. It further facilitates to perform quantitative tissue characterization as recently demonstrated in [22].

### Histological workup

After PC-CT data acquisition, the formaldehyde-fixed samples were cut into 5 mm slices according to histopathological workup guidelines. The slices were dehydrated in an ascending alcohol series before embedding in hot paraffin wax. After solidification, the paraffin blocks were cut into 5 µm sections using a standard microtome and sections were stained with hematoxylin and eosin using standard protocols.

### Correlation PC-CT data and histology

Absorption-contrast and PC-CT data were automatically co-registered and manually matched with corresponding histological slices.

## Results

PC- and absorption based CT data were successfully matched with corresponding histological sections based on characteristic macroscopic features and distribution of fatty tissue.

Macroscopic examination of the excised **tumor** **1** revealed a round shape with sharp borders and a white, gelatinous surface. The tumor reached the borders of the sample but appeared to be enclosed by a fibrous capsule. Histological workup confirmed the tumor as a fibroadenoma of 2.2 cm diameter, surrounded by fibrous-cystic mastopathy. Figure 1.D shows a representative hematoxylin-eosin stained histological slice of sample 1. The fibroadenoma appears with an edematous, hypocellular stroma interspersed with branched glandular complexes without atypia. The tumor boundaries are well defined against the surrounding tissue and the lesion is surrounded by a fibrous capsule. Figure 1.F shows the corresponding PC-CT image of sample 1: the fibroadenoma appears as a round structure with well-defined borders consistent with the findings of histopathology and ultrasound. The PC intensity is significantly lower compared to the surrounding breast tissue. The tumor itself appears of inhomogeneous contrast and is interspersed by filiform strands of high phase contrast, corresponding to strands of fibrous tissue in histology. Furthermore, small dark strands can be distinguished as a correlate of the branched ducts. The fibrous capsule of the fibroadenoma has a clear correlate consisting of different parallel layers of contrast-rich ring-shaped structures. The parallel layers as well as internal echogenic features are also clearly visible in the preoperative ultrasound (Figure 1.A). The surrounding breast tissue appears inhomogeneous and of higher contrast than the tumor, with inclusions of adipose tissue, seen in the upper right, that appear in dark grey corresponding to a low phase-contrast. Note that the ducts in Figure 1.D are artificially torn open during the histological workup. In the corresponding absorption-contrast image (Figure 1.E) adipose tissue appears in a dark grey corresponding to low absorption contrast values. Unlike in the PC-CT-images, there are no differences in contrast between the fibroadenoma and the surrounding breast tissue, and none of the internal structures mentioned above are visible. The tumor appears of homogenous contrast without differentiability of fine structures. The fibrous capsule is not as well defined as in the PC-CT image and may be reproduced only by following the lines of interspersed fat layers. Figure 1.G and H show the histograms of the whole 3D volume dataset of the absorption- and PC-CT images, respectively, with only two distinct peaks for adipose tissue as well as formalin, fibroadenoma and breast tissue in (G) and different grey levels for adipose tissue, formalin and fibroadenoma and surrounding breast parenchyma in (H).

The **tumor 2** was shelled out from the breast such that no surrounding breast tissue was adhering. Macroscopically, the tumor had a smooth, capsular surface. The cut face was pink and smooth, partially lobulated with white-grey septa of connective tissue between the lobuli. Histopathologic analysis (Figure 2.C) revealed a fibroadenoma consisting of tubular, branched glandular tubes as well as a stromal component of low cellularity. The glandular ducts consist of a two-row epithelium without atypia. The tumor extends close to the borders of the sample with only a few cell-layers in between. The nodular tissue components between the septa are well discernable in the PC-CT images (Figure 2.E). Interspersed strands of fatty tissue (bright in histology) appear as dark strands of low phase contrast. The tumor is of inhomogeneous phase-contrast with interspersed bright septa that correspond to strands of fibrous tissue in histology. The fine dark filiform structures correspond to the dilated ductuli that are characteristic of the fibroadenoma. In the absorption-contrast image (Figure 2.D) only two distinct grey shades (see histogram Figure 2.F) corresponding to adipose tissue as well as formalin, water and fibroadenoma can be differentiated whereas the histogram of the PC-CT data (Figure 2.G) reveals four distinct peaks for adipose tissue, water, formalin and fibroadenoma. There are no further contrast differences within the tumor visible in the absorption contrast image. In particular, the ductal structures are isocontrasted to the stromal component of the fibroadenoma.

Macroscopic examination of the excised **tumor** **3** revealed an ill-defined, irregular tumor of light-brown coloured surface surrounded by fibroglandular and fatty breast tissue. Microscopy (Figure 3.C) revealed a benign cystosarcoma phyllodes containing partially cystic and partially compressed ducts with a two-row epithelium. The stroma was hypercellular consisting of spindle-shaped cells. The well-defined, nodular components typical for fibroadenomas were not present. In the PC-CT slice (Figure 3.E), the tumor shows an intermediate contrast between the fatty tissue (dark) and the breast parenchyma (bright). Compared to the fibroadenomas, the tumor border is ill-defined and the typical branched ducts are missing. However, the differences in contrast between tumor and breast parenchyma in the PC-CT image allow a differentiation between the two tissue types. In the corresponding absorption-contrast image (Figure 3.D), the tumor and breast parenchyma appear in the same grey level, such that a differentiation is not feasible. Figure 3.F and G show the histograms of the absorption- and PC-CT datasets, respectively, with only two distinct peaks in (F) and different grey levels for adipose tissue, formalin & water, tumor and breast parenchyma in (G).

Macroscopic examination of the excised **tumor 4** showed an irregular tissue sample of firmly-elastic consistency with sparsely adhering adipose tissue. Microscopy (Figure 4.B) proved a fibroadenoma consisting of a lobulated stroma with low cellularity and rich in collagen fibers. Inside are multiple, partially ectatic, partially compressed branched ductuli with a regular two-row epithelium without atypia. Strands of fibrous tissue (arrows in Figure 4.B) are clearly reproduced in PC-CT (Figure 4.D). Dilated ducts are partially torn open and filled with formalin (arrowheads, Figure 4.B and D). In the corresponding absorption-contrast image (Figure 4.C), no inner structures within the fibroadenoma are differentiable. Figure 4.E and F show the histograms of the absorption- and PC-CT datasets, respectively. with distinct peaks for water, formalin and fibroadenoma in (F) and overlapping peaks for water, formalin and fibroadenoma in (E).

The excised **tumor 5** had a white surface and was surrounded by lobulated breast parenchyma. Histology (Figure 5.D) showed surrounding breast parenchyma with a well-defined nodular fibroadenoma, composed of collagenous, hypocellular stroma with multiple branched ductuli with typical two-row epithelium. The corresponding PC-CT section (Figure 5.F) reflects the histological features at high resolution. The tumor boundaries are well defined; thus, the tumor can be well differentiated from adhering fatty tissue (dark in phase contrast) and surrounding breast parenchyma. PC-CT reveals fibrous strands surrounding nodular parts within the tumor (see magnification view in 5.D). Even the characteristic branched, hairpin-shaped ducts (see arrow in Figure 5.D and F, dark violet in histology, dark grey in phase contrast) can be reproduced. An area where the ducts are more densely packed, indicated as a blue area in the left part of the tumor (arrowheads in Figure 5.D) corresponds to a region of darker overall grey levels in the PC-CT image (arrowheads in Figure 5.F).None of these inner structures are visible in the absorption contrast image (Figure 5.E). Figure 5.G and H show the histograms of the absorption- and PC-CT datasets, respectively, with only two distinct peaks for adipose tissue and formalin, water, fibroadenoma and breast tissue in (G) and different grey levels for adipose tissue, water, formalin and fibroadenoma & breast tissue in (H).

Macroscopic examination of the excised **tumor 6** revealed a well-defined mass of firmly-elastic consistency. Histological workup (Figure 6.B) revealed a fibroadenoma consisting of a hypocellular nodular stroma with interspersed branched, compressed filiform ducts. The tumor is surrounded by a thin pseudocapsule but focally reaches the resection margins. In the corresponding PC-CT image (Figure 6.D) the nodular components are well discernible. The strands of fibrous tissue in between are bright in the PC-CT image, corresponding to structures of high phase contrast. Dark filiform structures can be identified as dilated ducts. In the absorption-contrast image (Figure 6.C) the internal components of the fibroadenoma are not discernible. Only few dark spots corresponding to sparsely adhering adipose tissue can be differentiated from the otherwise homogeneous grey level of formalin and fibroadenoma. The histogram of the absorption-contrast CT dataset (Figure 6.E) shows only two distinct peaks for adipose tissue and formalin, water & fibroadenoma, whereas the histogram of the PC-CT dataset (Figure 6.F) reveals four distinct peaks for these structures.

Macroscopically, the cut surface of the excised **tumor 7** was whitish with firmly-elastic consistency. Histology (Figure 7.C) revealed a fibroadenoma with typically compressed, filiform ducts. The fibroadenoma was surrounded by a pseudocapsule and adhering adipose tissue. PC-CT (Figure 7.E) revealed the typical branched ducts within the fibroadenoma. In the corresponding absorption-contrast image (Figure 7.D), no inner structures of the fibroadenoma are visible. Figure 7.F and G show the histograms of the absorption- and PC-CT datasets with three peaks for adipose tissue, water and formalin & fibroadenoma in (F) but four distinct peaks for each of the structures in (G).

Inspection of the excised **tumor 8** revealed a whitish, firmly-elastic tumor. Histology (Figure 8.C) showed breast parenchyma with fibrotic changes and interspersed gland lobules. The stroma showed an enhanced proliferation and hyalinization, compatible with a pseudoangiomatous stromal hyperplasia (PASH). The PASH was confirmed by immunohistochemistry. In contrast to the fibroadenomas, the PASH shows now circumscribed growth and no clear demarcation. The PC-CT image (Figure 8.E) reveals areas of hyalinized stroma (bright) and ductal strucutes that are more loosely packed and less regular compared with the fibroadenomas. In the corresponding absorption-contrast image (Figure 8.D) no inner structures within the excised tissue sample are visible. The histograms (Figure 8.F and G) show only two distinct peaks for the absorption-contrast dataset but four peaks for adipose tissue, water, formalin and breast tissue for the PC-CT dataset.

Macroscopically, the excised **tumor 9** was of firmly-elastic consistency with a whitish surface. Histology (Figure 9.B) revealed a fibrous mastopahy with focal hyperplasia without atypia. The pseudocapsular surface prescribed above as a typical feature of the fibroadenomas is missing. No circumscribed nodular neoplasia is found, the mastopathy has no sharp demarcation. In the PC-CT image (Figure 9.D), the mastopathic area is well discernible from the adhering adipose tissue but there is no capsular surface and no nodular growth as described for the fibroadenomas. Few strands of fibrous tissue can be identified as well as ductal structures. However, both are not as regularly arranged as previously seen in the fibroadenomas. In the absorption-CT image (Figure 9.C), besides the adipose tissue and the mastopahy, there are no inner structures discernible. The histograms (Figure 9.E and F) reveal only two distinct peaks for the absorption CT dataset but four distinct peaks for the PC-CT image, corresponding to adipose tissue, water, formalin and mastopathy/breast parenchyma.

## Discussion

Several ex-vivo studies on tumor-bearing breast samples using highly-brilliant synchrotron sources have previously indicated that PC-CT provides excellent soft-tissue contrast and improved visibility of tissue fine structure formerly restricted to histopathology [15], [16]. PC-CT accurately depicts fine collagen strands, microcalcifications [15], strands of diffusely infiltrating lobular carcinoma [16] as well as the 3D outlines of ductal carcinoma in situ [18].

Fibroadenomas are the most frequent benign solid breast lesions. Highly specific diagnosis is crucial for a differentiation from potentially malignant lesions and thereby for justifying a conservative therapeutic approach. Due to different therapeutic approaches for fibroadenomas and the potentially malignant phyllodes tumor as well as carcinomas with benign imaging characteristics, a highly specific diagnosis of fibroadenoma allowing a conservative approach is of high relevance. Ultrasound and mammography show restricted specificity for fibroadenomas [7] and even core biopsy may miss relevant diagnostic findings, especially in large tumors [23], [24].

In this study on grating-based PC-CT of fibroadenomas using a polychromatic X-ray source, we show that grating-based PC-CT is – in contrast to absorption-based imaging - able to correctly depict the fine structure of fibroadenomas and to differentiate between tumor and surrounding breast parenchyma. Fibrous strands as well as the typical hairpin-shaped, branched ducts are visible using grating-based PC-CT technology. Breast samples containing a cystosarcoma phyllodes and benign breast changes mimicking fibroadenomas in preoperative ultrasound served as control. The cystosarcoma phyllodes in contrast to the fibroadenomas is lacking the sharp borders and pseudocapsular surface as well as the typical branched ducts. Both features and the lack thereof can be reproduced in PC-CT. The mastopathic area mimicking a fibroadenoma and a pseudoangiomatous stromal hyperplasia in contrast to the fibroadenomas show now circumscribed growth and no clear demarcation. PC-CT was able to depict fine structures and the characteristic growth patterns in analogy to histopathology. However, for an unequivocal diagnosis, histology remains the gold standard offering a significantly higher resolution and providing additional diagnostic tests such as immunohistochemistry.

In a first ex-vivo study, we have previously analyzed the diagnostic value of grating-based PC-CT using a polychromatic, laboratory X-ray source and have found that certain tissue features like dilated ducts, compact growing tumor clusters as well as regions of fibrous tissue are visualized in PC-CT but remain unseen in the corresponding absorption-contrast images taken at the same setup [17]. In accordance with these findings, fibrous strands within the fibroadenomas had a higher phase contrast compared to the surrounding tissue and regions of higher cellularity (i. e. the epithelial cells of the ducts) were of a lower phase contrast. These additional features might have the potential to improve the current breast imaging modalities.

Despite great technical improvements, the positive predictive value of screening mammography remains low due to a considerable rate of false positive findings [25]. The recall rate as well as the percentage of invasive diagnostics is consequently high in women undergoing their first screening mammography [25]. In these diagnostically unclear cases, PC-CT might serve as an additional tool and spare women from recall and invasive procedures.

In an ex-vivo setting, grating-based PC-CT might assist during histopathological workup. During routine histopathological workup of large tumors, only representative and macroscopically suspicious tissue sections are analyzed. This suggests that in large and inhomogeneous tumors, tissue sections of diagnostic relevance may be missed [24]. Here, grating-based PC-CT might help to identify diagnostically relevant parts within large tumors by offering a 3 D view of the entire tumor.

One major limitation of the currently used grating-based PC-CT setup is the high estimated dose applied. Our system is a not dose-optimized experimental setup; therefore the dose was not prospectively recorded. Rough calculations (see [17]) lead to an estimated total dose of several Gy, exceeding the maximum dose recommended for two-projection mammography by three orders of magnitude. However, PC-CT of whole breasts at synchrotron sources has been performed at clinically acceptable doses [26], [27]. Furthermore, advanced reconstruction algorithms allow a dose reduction in PC-CT of breast abladates by up to 74% [28].

Further limitations of our study include the small number of samples (n = 9) and the necessity of data acquisition after formalin fixation. Furthermore, due to modifications of the experimental setup during the study, not all samples were analyzed with the same imaging parameters. In particular, different gratings that were constantly advanced in quality were used and the distances between the gratings were adjusted in order to achieve an increased visibility and consecutively a higher contrast-to-noise ratio. The increase in quantum efficiency led to reduction of unnecessary dose and an increase in photon statistics. These changes led to a significantly reduced number of projections, exposure time and noise while maintaining constant image quality.

## Conclusions

The results presented here exemplify on the basis of a frequent solid breast tumor that PCI offers totally new imaging criteria that might complement breast diagnostics. We show here that PC-CT has theoretically the potential to improve breast imaging. Challenges to be overcome before the clinical implementation of the technique will be dose-reduction, integration into existing imaging systems as well as extensive evaluation in a blinded setup. Assuming that the technology can be successfully transferred to a clinical setting, grating-based PC-CT might serve as a complementary method for in-vivo and ex-vivo breast imaging.
